# Age Related Incidence and Early Outcomes of Hip Fractures: A Prospective Cohort Study of 1177 patients

**DOI:** 10.1186/1749-799X-6-5

**Published:** 2011-01-24

**Authors:** Anand Pillai, Vivek Eranki, Ravikiran Shenoy, Mahar Hadidi

**Affiliations:** 1 Department of Orthopaedics and Trauma, Wishaw General Hospital, Lanarkshire UK; 2 Department of Orthopaedics and Trauma, The Queen Elizabeth Hospital, South Australia, Australia

## Abstract

**Introduction:**

Associated with the increase in the aging population, there is an increase in the incidence of hip fractures worldwide. Outcome following such fractures is affected by age of the patient. This study aims to assess the incidence and early outcome of hip fractures, comparing between different age groups.

**Methods:**

Data of hip fractures collected over a period of five years was analysed. Patients were divided into three groups, group A (patients under the age of 64), group B (patients between 65 and 84 years of age), and group C (patients over the age of 85).

**Results:**

Of the 1177 patients included in the study, there were 90 patients in group A, 702 patients in group B and 385 patients in group C. There was a female preponderance across all age groups, and this increased as age advanced (p < 0.0001). A significantly larger number of older patients lived alone and needed aids to walk before the injury (p < 0.0001). There was no significant difference in the type of fracture across the three groups (p = 0.13). A higher proportion of the elderly with intracapsular fractures were treated by replacement arthroplasty. Older patients who had internal fixation of intracapsular fractures had a better walking ability at 4 months. The overall deterioration in mobility was greater in older patients (p < 0.0001). Mortality was higher in older patients.

**Conclusions:**

Hip fractures are more common among females irrespective of age group. Older patients have a higher mortality and a greater deterioration of walking ability after such injuries. Internal fixation of intracapsular fractures have demonstrated satisfactory early outcome in the immediate period. This could be attributed to retention of native bone, better propioception and shorter operation time.

## Introduction

The United Kingdom has a population of over 60.2 million with adults over the age of 65 forming 16% part of the population (10 million) [1]. Throughout the world it is predicted that the total number of hip fractures will increase from 1.26 million in 1990 to 2.6 million by the year 2025 and to 4.5 million by the year 2050 [2]. With the life time risk for a woman of sustaining a hip fracture being greater than that for developing a breast carcinoma[3,4], this fracture has gained an important place in terms of monitoring preventive and therapeutic measures for osteoporosis and falls. Earlier studies have reported a higher mortality attributable to the fracture with greater reduction in life expectancy in the younger age group and males compared to patients in the older age group and females [5,6]. The pattern of hip fracture [7-9] and the risk of social deterioration [10] are primarily determined by the age of the patient.

The aim of this study was to assess the affect of age on the incidence, fracture pattern, management and outcome of hip fractures in different age groups.

## Materials and methods

We analysed data on hip fractures collected prospectively over a period of five years at Wishaw General Hospital, Lanarkshire Scotland. This is a typical district general hospital which is the secondary referral centre for a population of approximately 200,000 people. Demographic details, pre- operative, intra operative and post operative details of these patients were collected. Patients were followed up for up to 4 months following the fracture. For the purpose of the study we divided the patients into three groups; group A, those aged 64 or less; group B, those between 65 and 84 and group C, those above the age of 85.

The type of surgery and post operative care determined by the type of fracture, age, co-morbid medical status and general level of mobility. The data was analysed using the SPSS 11.0 (SPSS inc, Chicago, Illinois). Variables between groups were compared using the chi square test at 95% confidence interval with p < 0.05 considered as significant.

## Results

During the five year period 1177 patients were admitted with hip fractures. There were 90 patients below the in group A (<64 years), 702 patients in group B (65-84) and 385 patients in group C*(> 85 years)*.

In Group A (n = 90), hip fractures were seen more commonly in females (71.1%). 90% of patients came from their own home and 71% living with family or friends. 67.4% of the patients were able to walk without any aids. There was a roughly equal distribution of intracapsular (51.1%) and extracapsular fractures (48.9%). 67.4% of patients with intracapsular fractures were treated by internal fixation and the remaining (32.6%) were treated by total or hemi arthroplasty. None of the patients were treated non-operatively. At 4 months 76.7% of patients were living in their own home and 23.3% were able to walk without any aids. The re-operative rate within the first 4 months was 6.7%. Mortality rate at 4 months was 12.2%.

In group B (n = 702), hip fractures were seen more commonly in females (77.8%). 65% of patients came from their own home and a greater proportion of patients compared to group A living alone (38.6%). 48.6% of the patients were able to walk without any aids. 53.6% had intracapsular fractures with 46.4% having extracapsular fractures. Of the patients with intracapsular fractures, 73.1% of patients were treated with a hemi or total arthroplasty and 26.9% had internal fixation. 4.5% of patients were treated non-operatively. By four months, 48.3% of patients were living in their own home and 7.7% were able to walk without aids by four months. There was a 4.4% re operation rate within 4 months. Mortality rate at four months was 20% with 56.2% of the patients treated non operatively.

In Group C (n = 385), hip fractures were seen more commonly in females (87%). 44.2% of patients came from their own home and 44.9% were living alone. 29.9% of the patients were able to ambulate without aids. There was an approximately equal distribution of intracapsular (48.5%) and extracapsular (51.5%) fractures. 79.1% of patients with intracapsular fractures were treated by a hemi or total arthroplasty. 4.2% of patients had non operative management. At 4 months 22.1% of patients were living in their own home and only 1.8% managed to walk without any aids. There was a 5.4% re operation rate within the first four months. Mortality rate at 4 months was 30.7% with 81.3% of the patients treated non operatively.

These results are summarised in tables 1 and 2.

**Table 1:** Summary of results

	Group A (<64 yrs)	Group B (65-84 yrs)	Group C (>85 yrs)
	
	*N = 90*	*N = 702*	*N = 385*
	
	**No**.	**No**.	**No**.
Male	26	156	50

Female	64	546	335

Pre op residence- own home	81	456	170

Pre op walking without aids	60	341	115

Intracapsular fractures	46	376	187

Extracapsular fractures	44	326	198

Internal fixation	75	405	217

Replacement arthroplasty	15	275	152

Non-operative	0	32	16

In hospital death	1	39	35

Living at home (4 months)	69	339	85

Walking un aided (4 months)	21	54	7

Total death in 4 months	11	140	118

**Table 2:** Summary of results of operated intracapsular fractures

	Group A (<64 yrs)		Group B (65-84 yrs)		Group C (>85 yrs)	
	N = 46		N = 357		N = 177	
	
	Internal fixation	Replacement arthroplasty	Internal fixation	Replacement arthroplasty	Internal fixation	Replacement arthroplasty
	
	*N = 31*	*N = 15*	*N = 96*	*N = 261*	*N = 29*	*N = 148*
	
	**No**.	**No**.	**No**.	**No**.	**No**.	**No**.
Pre injury living at home	30	12	68	169	9	69

Pre injury walking unaided/one stick	28	13	84	206	20	105

Living at home in 4 months	26	12	56	130	8	36

Walking unaided at 4 months/one stick	20	10	44	79	9	14

Re operations	4	1	6	12	1	11

Total death	2	1	13	39	4	47

## Discussion

Hip fractures are reported to be more common in females and the elderly [1,11,12]. In this series the fracture was seen more commonly in females across all three age groups. This female preponderance was found to significantly increase with advancing age (p < 0.0001). This could perhaps be attributed to the higher female to male ratio in the general population as age increases and lower bone density (BMD) in women compared with men [13]. Group C demonstrated a lower number total number (n = 385) compared to group B. Since the average life expectancy in Scotland is 75.3 years for males and 80 years for female [1], it could be argued that patients in group C have outlived their normal life expectancy hence causing a reduction in the total number of people in this group in the general population with a resulting lower number of patients developing a hip fracture.

**Figure 1:**
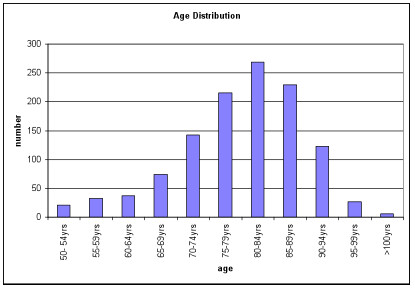
Age distribution of hip fracture

Proportionally, majority of patients with neck of femur fractures belong to group C (Figure 1). A significantly lower number of older patients were resident in their own home and were able to walk alone outdoors at the time of fracture (p < 0.0001). Compared to groups A and B, a higher proportion of the patients in group C needed aids to mobilize (p < 0.0001). This could have a bearing on the increase in number of patients developing a hip fracture in the elderly. A previous meta-analysis of 16 case series has demonstrated that in females between the ages of 50 and 60, and in men over the age of 70, intracapsular fractures are more common than trochanteric fractures [7]. Another study has shown the proportion of hip fractures that occurred in the trochanteric region to rise steeply with age among Caucasian women compared to other demographics and males [8]. Hip fracture pattern is more related to the trochanteric and femoral neck BMD and proximal femoral geometry rather than age, gender, fall characteristics and body habitus [14-16]. In our study there was no statistically significant difference in the number of intra and extracapsular fractures between the three groups (p = 0.13). 5% of patients with intracapsular fractures in groups 2 and 3 were treated non-operatively owing to their co-morbidity. A higher portion of the intracapsular fractures were treated by replacement arthroplasty in the older age groups (32.6%, 69.4% and 79.1% respectively, p < 0.0001). We compared the change in residential status and walking ability between those who had internal fixation and those who had replacement arthroplasty for intracapsular fractures between the three groups at 4 months. Results are summarised in table 2.

The type of fixation varied based on the patient group. In Group A, 67% of the patients underwent internal fixation while 33% underwent hemi/total arthroplasty. In Group B, the rates of internal fixation dropped to 27% and further to 16% in Group C. Patients in Group B and Group C, who had internal fixation fared better at 4 months compared to those who have hemi/total arthroplasty with no statistically significant difference in re operation rates. It has been reported that over a longer period of follow up younger patients with a replacement arthroplasty have a better walking ability with lower re operation rates [17,18]. However we do not have any longer term follow up data on our patients to verify this.

Perioperatively (in-hospital) the mortality rate were 1.1% in Group A, 5.6% in Group B and 9.0% in Group C. The mortality rate rose to 12.2% in Group A, 19.9% in Group B and 30.6% in Group C at 4 months (p < 0.0001). There was a significantly higher mortality associated with hip fractures with increasing age. Between Group A and C, this represents a 900% in increase in peri-operative and 250% increase 4 months post operative mortality. Our results compare well with other reports [19,20]. A recent study suggests that the one year mortality rate in patients with hip fractures over the age of 95 is no worse than in patients below this age [21]. There is also no significant increase in mortality attributable to the hip fracture in the elderly compared to the general population of the same age [22].

Re-operation rate in group B was 6.3% for those who had internal fixation compared with 4.6% for those who had a hemi/total arthroplasty (p = 0.59). While, in group C was 3.4% and 7.4% following internal fixation and replacement arthroplasty respectively (p = 0.69).

Most of the functional recovery after a hip fracture occurs by 4 months [23]. In our study 85.2% of patients in group A who came from their own home returned home by four months, compared to 74.3% and 50% in group B and C respectively (p < 0.0001). Among patients who were independently mobile or walking with one stick before the injury, 66.2% in the group A regained this level of mobility by four months compared to 40.1% and 16.7% in group B and C respectively (p < 0.0001). This shows a significant deterioration in both, walking ability and residential status in the elderly who sustain these fractures. Age is reported to be a significant variable affecting functional recovery after hip fractures [24], although cognitive function, presence of co-morbid factors and pre- injury function in terms of activities of daily living have a significant impact in recovery [25]. We have not evaluated the role of these additional factors on outcome however, it would be safe to assume deterioration in these factors with age.

From group A, 86.7% of patients who have been living in their own home for greater than 4 months had internal fixation and managed to return to their home. All of the patients who came from their own home and had a replacement arthroplasty were back at home by 4 months (p = 0.3). 71.4% of patients regained their mobility after internal fixation, compared to 76.9% following hemi or total arthroplasty (p = 1.0). Type of surgery did not make a statistically significant difference in these outcomes and reoperation rate (p = 0.52). In group B, among the patients who had an internal fixation of the intracapsular hip fracture, 82.4% returned to their home by four months compared to 76.9% following a replacement arthroplasty (p = 0.39). 52.4% of patients who were independently mobile prior to their fracture regained mobility after internal fixation. This value was 38.3% among those who had a replacement arthroplasty (p = 0.028). Hence patients who had internal fixation had a statistically significant improved walking ability compared to those who had replacement arthroplasty in this group. In the Group C, of patients, 88.9% of patients returned home following an internal fixation compared to 52.2% following a replacement arthroplasty (p = 0.037). 45% of patients who were walking independently or with one stick managed to do so at 4 months following internal fixation, whereas following a replacement arthroplasty this figure was only 13.3% (p = 0.0008). Again type of surgery made a statistically significant difference in outcome, with those having internal fixation faring better at four months.

## Conclusions

Hip fractures were more common among females across all age groups. There was no significant difference in fracture patterns between the groups. A higher mortality and a greater deterioration of walking ability were noted among older patients. A larger proportion of older patients with hip fractures were unable to return home. In patients over the age of 65, at 4 months, a better walking ability and lower re operation rate was found after internal fixation compared to replacement arthroplasty. This variation was not seen in younger patients.

## Competing interests

The authors declare that they have no competing interests.

## Authors' contributions

AP, VE and RS designed the study, accumulated data, analysed data, drafted manuscript and MH supervised the entire study. All authors read and approved the final manuscript.
